# Continuous Biopotential Monitoring via Carbon Nanotubes Paper Composites (CPC) for Sustainable Health Analysis

**DOI:** 10.3390/s23249727

**Published:** 2023-12-09

**Authors:** Seunghyeb Ban, Chang Woo Lee, Vigneshwar Sakthivelpathi, Jae-Hyun Chung, Jong-Hoon Kim

**Affiliations:** 1 School of Engineering and Computer Science, Washington State University, Vancouver, WA 98686, USA; seunghyeb.ban@wsu.edu; 2 Department of Mechanical Engineering, University of Washington, Seattle, WA 98195, USA; cwl20@uw.edu (C.W.L.); jae71@uw.edu (J.-H.C.)

**Keywords:** carbon nanotube paper composite, biopotential measurement, EOG, ECG, EEG

## Abstract

Skin-based wearable devices have gained significant attention due to advancements in soft materials and thin-film technologies. Nevertheless, traditional wearable electronics often entail expensive and intricate manufacturing processes and rely on metal-based substrates that are susceptible to corrosion and lack flexibility. In response to these challenges, this paper has emerged with an alternative substrate for wearable electrodes due to its cost-effectiveness and scalability in manufacturing. Paper-based electrodes offer an attractive solution with their inherent properties of high breathability, flexibility, biocompatibility, and tunability. In this study, we introduce carbon nanotube-based paper composites (CPC) electrodes designed for the continuous detection of biopotential signals, such as electrooculography (EOG), electrocardiogram (ECG), and electroencephalogram (EEG). To prevent direct skin contact with carbon nanotubes, we apply various packaging materials, including polydimethylsiloxane (PDMS), Eco-flex, polyimide (PI), and polyurethane (PU). We conduct a comparative analysis of their signal-to-noise ratios in comparison to conventional gel electrodes. Our system demonstrates real-time biopotential monitoring for continuous health tracking, utilizing CPC in conjunction with a portable data acquisition system. The collected data are analyzed to provide accurate heart rates, respiratory rates, and heart rate variability metrics. Additionally, we explore the feasibility using CPC for sleep monitoring by collecting EEG signals.

## 1. Introduction

Skin-based wearable devices in healthcare monitoring have drawn much attention with the recent development of soft materials and thin-film technologies. The wearable devices require biocompatible and flexible electrodes for safe and seamless integration with human skin to achieve high performance. Prior studies used conventional Ag/AgCl gel electrodes to achieve high-fidelity recording. However, the gel used on the skin often causes several issues, such as poor breathability, skin irritation, and loss of performance for long-term monitoring coupled with drying [1]. Rigid metallic electrodes, such as Ag/AgCl and Au, have also been used as dry electrode materials to acquire bio-potential despite the mechanical mismatch between the rigid electrodes and the soft human skin [2,3,4].

To address the challenges, the field of flexible electronics has been dedicated to the creation of stretchable and flexible electrodes that conformally contact the skin [5]. However, the fabrication of these electrodes requires micro-patterned mesh structures and employs a variety of polymers to attain attributes such as flexibility, stretchability, and low density. While microfabrication can realize large-scale manufacturing, it requires high-end, expensive equipment within a cleanroom or dedicated facility. Various printing technologies, including screen printing, inkjet printing, and aerosol jet printing, are also applied to fabricate electrodes and sensors. These printing methods are more facile and designable than microfabrication. However, printing technologies have limitations when it comes to scalability.

The use of paper as a substrate for wearable electrodes continues to increase in interest due to its porous structure, low cost, and flexibility. Paper is also inexpensive, making it a good candidate for disposable electrodes. Also, flexibility is essential to conform to curved surfaces such as human skin. Another advantage of paper-based electrodes is scalability. The paper-based electrode fabrication process can be easily customized to its size and scaled quickly and inexpensively.

Flexible and lightweight paper can be used as biopotential electrodes with a coat of conductive materials. Carbon nanomaterials, such as carbon nanotubes (CNTs), graphene, and carbon black, can serve as the coating layer of paper, making it electrically conductive. However, their toxicity levels and effects on the human body, such as oxidation stress, increased skin cell count, glutathione depletion, and skin thickening, still remain a concern. To prevent these negative effects, the solutions using a composite of polymer material and carbon nanomaterials have been studied. PDMS, TPU, and PEDOT:PSS materials are elastic, flexible, and non-toxic. PDMS is an elastic and flexible material that is commonly used in biomedical applications due to its non-toxicity [6,7,8]. CNT/TPU composites offer exceptional stretchability, sensitivity, stability, and reliability. They can be produced using eco-friendly methods. These composites can be used to create sensors [9,10]. PEDOT:PSS/CNT composite material, which is a stretchable thermoelectric composite, has been designed to detect human motions. This material is low-cost, non-toxic, and flexible, gaining attention for its potential applications [11,12]. However, these composites are fabricated by the random dispersion of carbon materials in a polymer solution. It also still has direct skin contact with carbon materials, which is concerning due to their biocompatibility issues.

Here, we use paper as a substrate to make composite electrodes with CNTs for biopotential measurement. The CNT–paper composites (CPC) are then coated with various polymer materials such as PDMS, Eco-flex, PI, and PU. We can acquire biopotential safely using polymer-coated CPC electrodes while preventing direct skin contact. This provides greater enhanced biocompatibility than previous random dispersed conductive polymers. While conductive polymers acquire biopotential by the resistive and capacitive connection of the skin and electrode, the polymer layer on our electrode acts as a capacitive layer. Due to the large surface area of cellulose fibers and CNTs’ large intrinsic capacitance [13,14], the large capacitance of the coated CPC will have a low skin–electrode impedance for stable biopotential measurement [15]. 

The coated CPC electrodes are characterized using an electrooculography (EOG) system to compare the signal-to-noise ratios (SNRs) and analyze biopotential through polymer-coated CPC electrodes. The SNR values of PDMS- and Eco-flex-coated CPC electrodes are compared to the conventional gel electrode through the measurement of an EOG, electrocardiogram (ECG), and electroencephalogram (EEG). This ECG system allows for the collection of accurate heart rates, respiratory rates, and heart rate variability metrics for continuous health monitoring. Furthermore, the coated CPC electrode system can collect EEG data to show the possibility of developing a sleep monitoring system. The experimental result maintains a stable connection during a 30-min nap without any modification of the commercial monitoring device. With a simple cleaning for hygiene, the coated CPC electrode could be repeatedly used by six subjects. These CPC electrodes are mass-producible, reusable, and available for long-term use at home. The results show that the CPC electrode system can be an alternative to bulky and uncomfortable metal electrodes for measuring biopotentials. These simple methods also reduce the installation time.

## 2. Materials and Methods

### 2.1. Materials

CNT ink was made by an aqueous solution of multiwall carbon nanotubes (MWCNTs) (5 mg/mL; Nanostructured & Amorphous Materials, Inc., Houston, TX, USA). A 100 µm thick porous paper (Schott^®^ Hand Paper Towel) was used as a template. Silicone elastomers, including Eco-flex 00-30 and polydimethylsiloxane (PDMS, Sylgard 184), were obtained from Smooth-On and Dow Corning, respectively. Polyimide (PI) ink, named PI-2545, was prepared from HD microsystems. Also, polyurethane (PU) was obtained from the Minwax company. Silver ink (Kayaku Advanced Materials AG-510 Silver Conductive Ink, Westborough, MA, USA) was prepared with a brush to the paper’s ends to create contact pads. Fast-drying silver paint was purchased from Ted Pella.

### 2.2. Fabrication of CPC Electrodes

**Figure 1:**
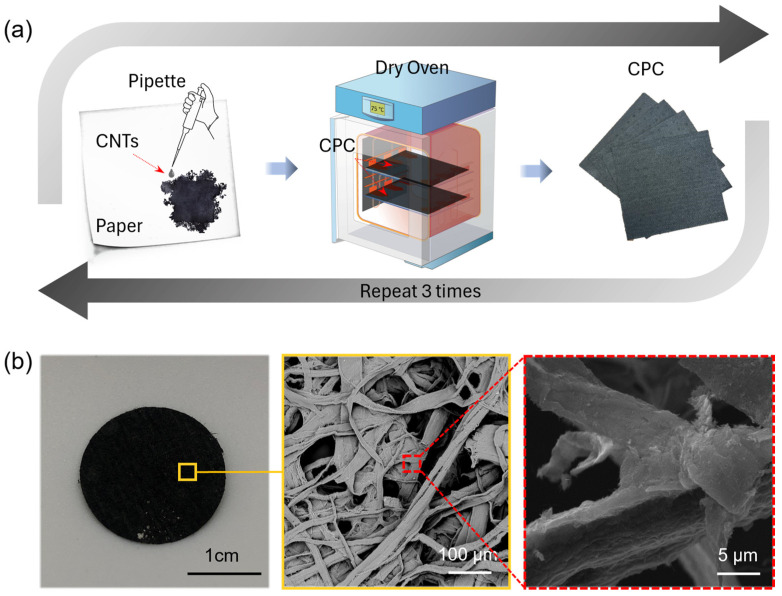
CPC manufacturing method and characterization. (**a**) Schematic of the CPC manufacturing process. (**b**) Photo of CPC electrode and SEM images showing cellulose fibers coated with CNTs.

Figure 1a shows the summary of the fabrication procedure for CPC electrodes. First, an aqueous solution of multiwall carbon nanotubes (MWCNTs) was sonicated in a 10 mL water-based solution (sodium dodecyl sulfate; SDS; 1%) for 3 h. Then, 400 µL of MWCNT solution was pipetted onto one side of the porous paper (60 × 60 mm^2^). A total of 200 µL of MWCNTs solution was pipetted onto another side of the paper to avoid over-wetting. The coated paper was cured in an oven for 15 min at 75 °C. We repeated the coating with MWCNTs solution and cured twice more. Figure S1 presents the step-by-step CPC fabrication procedure. Finally, we cut the paper into the desired size and applied the silver paste as a contact pad on the coated paper, as shown in Figure S2.

### 2.3. Validation of Biopotentials with an EOG, ECG, and EEG System

The CPC electrodes were coated with various polymer materials, including PDMS, Eco-flex, PI, and PU, to collect the biopotentials. We performed EOG tests to compare the SNRs for the conventional gel electrodes and the coated CPC. To measure EOG signals, we used two target electrodes, and a conventional gel electrode (Red Dot electrodes, 3M) for a common grounding electrode, with BioRadio as a commercial biopotential acquisition system (Great Lakes NeuroTechnologies, Independence, Cleveland, OH, USA, Figure S3). Two electrodes (Channel 1+ and Channel 1−) were positioned 1 cm to the side of each eye. A common grounding electrode was placed on the left mastoid (behind the left ear as shown in Figure S4) [16]. By comparing the SNRs and resistance values, we decided to use PDMS-coated CPC electrodes for measuring ECG and EEG signals. The ECG data collection was also performed via BioRadio with two measurement PDMS-coated CPC electrodes and one conventional gel electrode as a reference electrode. To measure ECG signals, target electrodes were attached to the left side of the chest, and one conventional gel electrode was placed on the upper chest area. The collected EOG and ECG data were wirelessly transmitted to a computer for analysis. In the case of ECG signal processing, the acquired ECG signal was filtered by the Pan–Tompkins algorithm to distinguish R-peaks in the signals and suppress other peaks [17]. The performance of the EEG measurement was tested with a commercial sleep monitoring device (SOMNOtouch RESP, SOMNOmedics, Figure S5). This device had an EEG/EOG combined sensor module consisting of two EOG electrodes: one EEG electrode, which acted as a reference electrode, and a ground electrode. The ground and reference channels were always attached to the gel electrodes (ArboTM H124SG, Kendall, Mansfield, MA, USA). On EOG/EEG channels, the PDMS-coated CPC and gel electrodes were compared through non-simultaneous measurements because there was only one channel available at each measurement point. The skin was cleaned with Nuprep skin preparation gel before attaching the electrodes. The coated CPC electrodes were simply attached to the skin with medical tape (NexcareTM, 3M, St. Paul, MN, USA). The recordings were stored on the device’s internal memory and transferred to the computer after recording. 

### 2.4. Data Acquisition via Commercial Medical Devices

Commercial medical devices, such as BioRadio and SOMNOtouch RESP (SOMNOtouch), were used to detect biosignals such as an electrooculogram (EOG), electrocardiogram (ECG), electromyography (EMG), and electroencephalogram (EEG). Wireless data collection via BioRadio made it possible to acquire EOG or ECG through multiple channels (four channels are available). The device size was 10 × 6 × 2 cm^3^ (Figure S3). BioRadio included both Bluetooth Low Energy and classic Bluetooth. The transmission range of BioRadio was around 30 m, and the sampling rate was 250 Hz [18]. The battery time was up to 8 h, which was sufficient for daily use. Detailed specifications of BioRadio are shown in Figure S3. SOMNOtouch was a portable polysomnography device that was developed for sleep monitoring. The device size was 8.4 × 5.6 × 1.8 cm^3^ (Figure S5). SOMNOtouch had 11 channels, and EEG/EOG/ECG channels were tested with coated CPC electrodes. EOG channels were measured at 128 Hz without filtering. The ECG channel was measured at 256 Hz with a 0.3 high-pass filter and a 50 Hz notch filter. The EEG channel was measured at 256 Hz with a 1 Hz high-pass filter and a 35 Hz low-pass filter. The manufacturer also provided a post-processing program for automatic sleep analysis. Detailed specifications of SOMNOtouch are shown in Figure S5.

### 2.5. Signal-to-Noise Ratio

The coated CPC electrodes were characterized by computing the SNRs of EOG and ECG signals. EOG signals and ECG signals were acquired for 5–8 s via BioRadio. SNR measurements followed prior studies [5]. To compute the SNR values of the acquired EOG signal, measurement of EOG signal amplitude and removal of the average value of the EOG signal was conducted by using the following equation: $SNR\left(dB\right)=10\;{Log}_{10}\left(\frac{RMS\_signal}{RMS\_noise}\right)$. Also, the SNR values of the acquired ECG signal were calculated using the average amplitude of the QRS complex that included Q-, R-, and S waves and the average noise amplitude using the above SNR (dB) equation.

## 3. Results

### 3.1. Overview of CPC System Fabrication

Figure 1 summarizes the overview of the CPC electrode fabrication method for biopotential measurement. Figure 1a shows the step-by-step procedure for the fabrication of CPC electrodes. The fabrication method of CPC electrodes involved the coating of porous paper with an aqueous solution of MWCNTs and then curing it in an oven at 75 °C for 15 min. This process was repeated three times. To examine the surface of the paper coated with CNTs, we used a scanning electron microscope (SEM). The SEM photos show the cellulose fibers wrapped with CNTs (Figure 1b). We used a four-point probe method to ensure accurate measurements of the sheet resistance of CPC paper and eliminated the errors due to contact resistance. The average sheet resistance of the four CPC sheets was 420 Ω/□. Detailed data are shown in Table S1.

**Figure 2:**
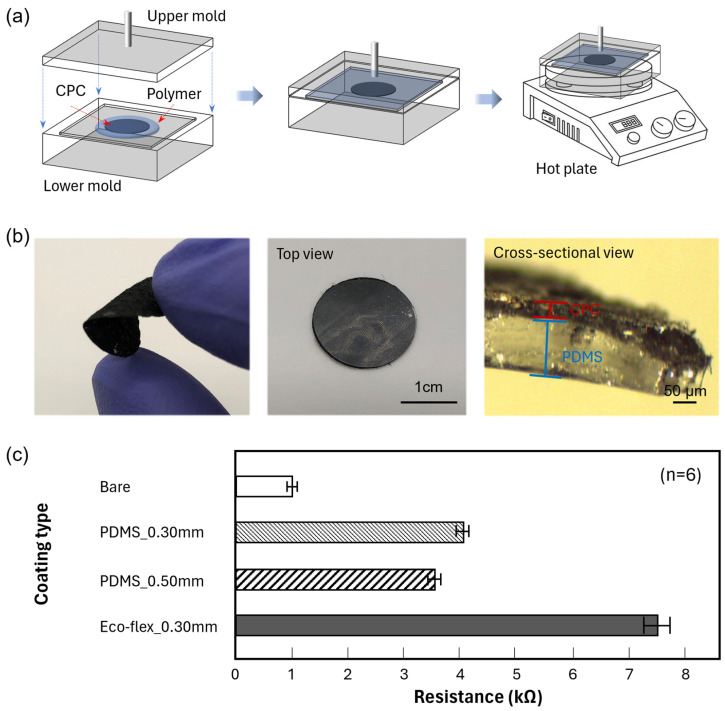
Polymer layer coating method and characterization. (**a**) Schematic of the polymer layer coating process. (**b**) Photos of the polymer-coated CPC electrode with the top view and the exploded cross-sectional view. (**c**) Comparison of resistance on a bare electrode and the polymer-coated CPC electrodes.

To avoid direct contact with the CPC electrode on the human skin, we covered the top side of the CPC electrodes with biocompatible polymer materials, including PDMS, Eco-flex, PI, and PU. As shown in Figure 2a, we made customized molds to produce thin polymer film with target thicknesses. The target thicknesses of the polymer film are 0.30 and 0.50 mm. With those customized molds, we could control the thickness of the biocompatible polymer layers on the CPC electrodes. CPC electrodes were placed on the mold and covered with each polymer. Once the mold was assembled, it was baked on a hot plate for curing. The curing temperature used on the hotplate varied depending on the polymer materials. The PDMS was cured at 150 °C for 30 min [19]. Eco-flex was cured at room temperature for 3 h. For faster curing, Eco-flex was heated to 120 °C for 15 min. PI required a high temperature of 200 °C in the oven for 20 min. Also, PU was cured at room temperature for 3 h. After curing, the mold was cooled at room temperature for 10 min (Figure S6). Figure 2b shows the flexibility of paper-based electrodes after the MWCNTs coatings [20], which was sufficient to accommodate human skin deformations. Also, the top and side views of polymer-coated CPC electrodes are presented in Figure 2b, showing that the polymer-coated CPC electrode was fully covered with PDMS.

Since the polymer material also penetrated the CPC electrode during the coating procedure, the resistance of the CPC electrode varied after the coating process. We measured the resistance at 8 mm intervals on the bottom side of the CPC electrodes to quantify the resistance change, as shown in Figure S7. Unlike CPC papers, the resistance of CPC electrodes was obtained by the two-probe method. Since the paper was trimmed to a small electrode shape and coated, it no longer met the thin-film assumption of the sheet resistance. PI and PU were fully absorbed into the CPC, making it challenging to achieve a consistent thickness as well as a stable resistance measurement. Therefore, we only presented the resistance values for Eco-flex and PDMS-coated CPC electrodes, which could control the polymer layer thickness and measure the resistance of the uncoated portions. In the case of the Eco-flex-coated CPC electrodes with a 0.5 mm thick coated layer, we excluded it from Figure 2c because it could not read the biopotential signals. As shown in Figure 2c, the bare CPC electrode resistance was 0.89 kΩ. The CPC electrode coated with Eco-flex (0.30 mm target mold: 7.63 kΩ) showed a relatively higher resistance compared to the electrode coated with PDMS (0.30 mm target mold: 3.83 kΩ and 0.50 mm target mold: 3.46 kΩ). Table S2 shows the detailed CPC electrode resistance. We also checked the resistance on the polymer-coated surface. PDMS and Eco-flex completely covered the top side of CPC electrodes, resulting in a non-conductive status by a digital multimeter (Fluke Corporation, Everett, WA, USA). However, the resistance for the top side of CPC electrodes coated with PI and PU was still measurable (Figure S8). 

### 3.2. Evaluation of the CPC Coating Layer via the EOG System

**Figure 3:**
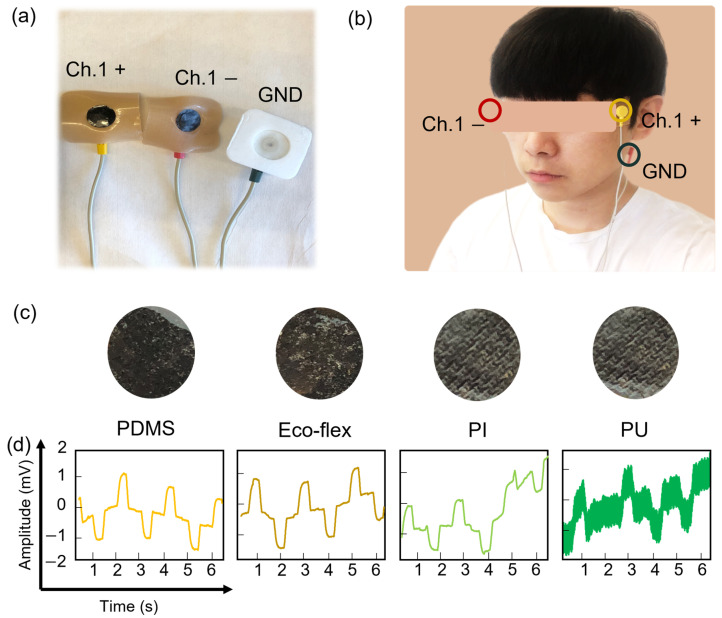
Characterization of the polymer-coated CPC electrodes. (**a**) Photos of two polymer-coated electrodes and one commercial gel electrode. (**b**) A picture of the face with electrodes attached. (**c**) Photos of polymer-coated CPC electrodes (PDMS, Eco-flex, polyimide, and polyurethane). (**d**) EOG signal comparison of polymer-coated CPC electrodes (single channel comparison).

To evaluate the CPC electrodes covered by various polymer materials for biopotential measurement, we collected EOG signals and compared SNR values. We used a single channel in BioRadio with 0.3 mm thick polymer-coated CPC electrodes, as shown in Figure 3a. BioRadio included Bluetooth Low Energy (BLE) and classic Bluetooth, and the sampling rate was 250 Hz. BLE was low-power and suitable for continuous health care monitoring, while classic Bluetooth had a higher data transfer rate and a more extended range for real-time data transmission. Including both options allowed users to choose the best type of Bluetooth connection for their specific needs. Two CPC electrodes were connected with Channel 1+ and Channel 1− snap electrode lead wires and placed 1 cm outside both eyes. A common grounding electrode connected with the GND snap electrode lead wire was placed behind the left ear, as shown in Figure 3b [16]. Figure 3c shows photos of coated CPC electrodes with various polymers. During the coating process, the CPC electrode did not completely absorb the PDMS and Eco-flex, resulting in most of the materials being coated on the surface of the CPC electrode. They maintained their adhesion properties, making it easy to attach the electrode to the skin. However, in the case of PI and PU, the CPC electrode fully absorbed these materials, resulting in a lack of adhesion on the surface, inconsistent coating quality, and unstable resistance values. The non-adhesive PU-coated CPC electrode was susceptible to ambient noise and users’ delicate movements. Also, users could be exposed to the CNTs due to inconsistent coating across the electrode. Thus, the use of PI and PU as coating materials might be limited due to the effects of carbon-based nanomaterials on human skin [21,22]. Furthermore, our previous work showed the skin biocompatibility of a gel electrode. As shown in Figure S9, the gel electrode caused skin irritation and temperature elevation after 4 h [23]. For comparison, we performed an EOG measurement using CPC electrodes coated with all four different polymer layers, as shown in Figure 3d. We compared SNR values to validate coating materials for detecting biopotentials. Detailed SNR values are shown in Figure S10. The Eco-flex-coated CPC electrode had a slightly higher SNR value than the PDMS-coated and PI-coated CPC electrodes. The coated CPC electrodes could be used to obtain biosignals [24]. However, the PU-coated CPC electrode could not be analyzed due to the noise. Also, PDMS had a higher moisture vapor transmission rate (MVTR) than Eco-flex, which was crucial for maintaining the adhesive force influenced by sweat emission [25]. Additionally, PDMS’s lower resistance than Eco-flex was advantageous for minimizing susceptibility to noise and motion artifacts during measurements [26,27]. Based on the results, we decided to use PDMS as the biocompatible coating material for our experiments. As shown in Figure S11, the graph compared EOG signals using CPC electrodes coated with PDMS and conventional gel electrodes. There was no significant difference between the two signals.

### 3.3. Characterization of the CPC Electrode System

**Figure 4:**
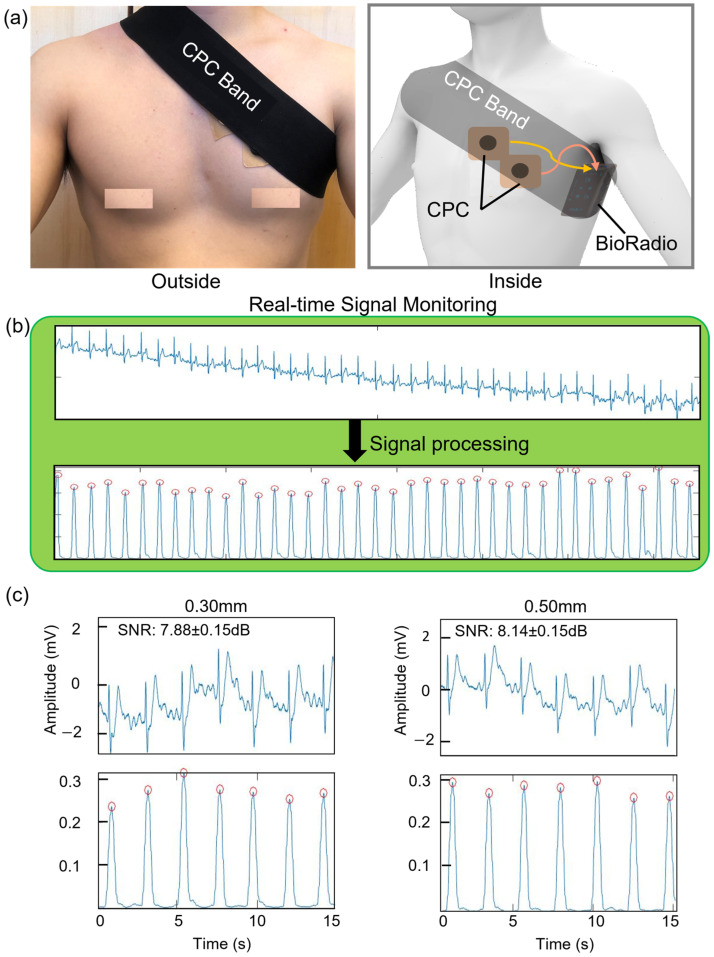
Overview of the CPC electrode performance demonstration test. (**a**) CPC system on the chest for ECG measurement: outside view (**left**) and inside view (**right**). (**b**) Illustration of signal processing of the ECG signals via the CPC system. (**c**) The signal plots describing the signal amplitude with results by PDMS-coated CPC electrodes.

Many wearable devices still rely on bulky sensors and wires, increasing noise levels in signal acquisition [23]. This study used the band-type platform to secure the CPC electrodes on the user’s skin to demonstrate portability. As shown in Figure 4a (left), the band-type platform covered the user’s chest and secured the CPC electrodes and wires to prevent movement from daily life. Figure 4a (right) shows the inside of the band-type platform. Two PDMS-coated CPC electrodes were mounted inside the band-type platform, and BioRadio was hanging on the back of the band. The band-type platform size was 8 × 100 cm^2^. We used a single channel in BioRadio with CPC electrodes embedded with PDMS. As with measuring EOG signals, we monitored real-time ECG signals and collected raw ECG data via BioRadio. As shown in Figure 4b, our signal processing algorithms based on the Pan–Tompkins algorithm analyzed the collected ECG raw data. The SNR values of the electrodes coated by biocompatible materials were measured by our signal-processing algorithms and were compared by thickness (0.30 and 0.50 mm). As shown in Figure 4c, the SNR value of the 0.30 mm CPC electrode (7.88 ± 0.15 dB) had a slightly lower SNR than that of the 0.50 mm CPC electrode (8.14 ± 0.15 dB). A 0.50 mm thick CPC electrode also showed the SNR value that could detect ECG sufficiently. The shirt fabric thickness ranged from 0.13 mm in thickness to 0.38 mm in average. Therefore, CPC electrode systems could be mounted on top of clothes to show the potential for user health monitoring applications. We also measured ECG signals using CPC electrodes coated by PDMS and conventional gel electrodes. As shown in Figure S12, there was no significant difference between the two ECG signals.

### 3.4. Signal-Processing Algorithms for Monitoring ECG and Applications

**Figure 5:**
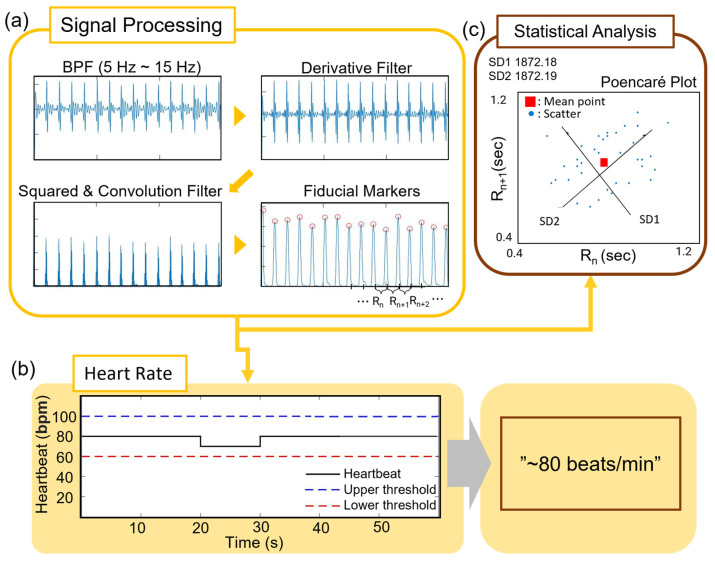
Development of the algorithm for heart rate variability detection. (**a**) Overview of data processing methods: HRV estimation process and (**b**) HR estimation process. (**c**) Statistical analysis with Poincaré Plot.

Figure 5 illustrates the complete data processing and analysis steps for estimating the ECG, heart rate (HR), respiration rate (RR), and heart rate variability (HRV). HRV was an indicator of the temporal variation between heartbeats. The R–R interval, defined as the duration between two adjacent R wave peaks, was used to measure the regularity of heartbeats and was crucial for arrhythmia diagnosis. To estimate HRV metrics, we developed an algorithm that calculated the standard deviation of the R–R deviation (SDRR), the root-mean-square of successive differences (RMSSD), the coefficient variation of the R–R variation (CVRR), and the Poincaré plot, which displayed the values of each pair of R–R intervals. Raw ECG data were first processed using a band-pass filter to eliminate baseline drift and motion artifacts, as shown in Figure 5a. Next, the Pan–Tompkins algorithm was used to detect the QRS complex and the heartbeat sequence. Subsequently, R-peaks were spline interpolated for RR estimation. To detect arrhythmia, the algorithm differentiated the time between R-peaks and the following R-peaks. The collected interval data were then processed to derive the HRV metrics (detailed equations are in Equation (S1)). The final output included clinical analysis data such as HR (beats/min) (Figure 5b), RR (breaths/min) (Figure S13), SDRR, CVRR, and the Poincaré plot (Figure 5c), which were statistical data for arrhythmia detection, demonstrating the performance of the CPC. The CPC band-type platform allowed for stable signal acquisition due to the ability to secure multiple CPC electrodes to the subject. Furthermore, this technology demonstrates the potential to facilitate the development of sports equipment that monitors workout performance, as it can be easily integrated into clothing.

### 3.5. EEG Measurement and Sleep Monitoring Application

**Figure 6:**
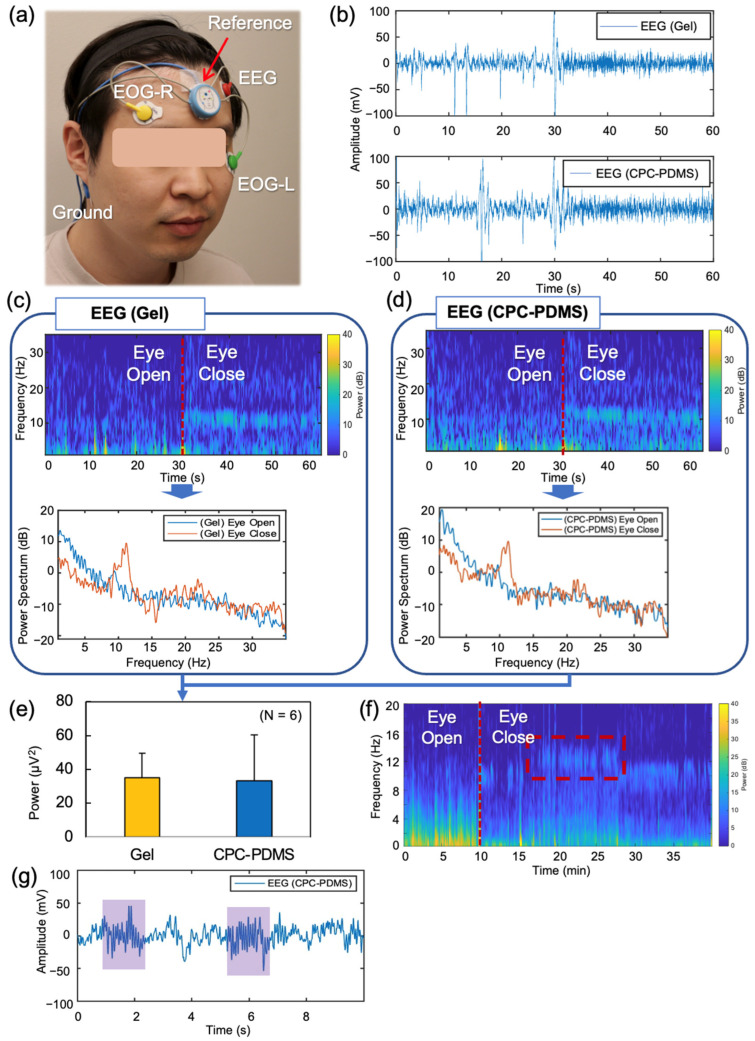
EEG validation of CPC electrodes. (**a**) A photo of electrodes attached to the subject. (**b**) EEG with gel electrode (**above**) and PDMS-coated CPC (CPC-PDMS) electrode (**below**). (**c**) Spectrogram and power spectrum of the EEG with the gel electrode. Red dotted lines represent the eye-closing state. (**d**) Spectrogram and power spectrum of the EEG with the PDMS-coated CPC electrode. (**e**) Average alpha band power difference of eye close to eye open from the six subjects. (**f**) Spectrogram of the EEG having a short nap with the PDMS-coated CPC electrode. (**g**) Sleep spindles detected during the short nap on (**f**).

To measure EEG signals, we used the commercial device SOMNOtouch and 0.3 mm thick PDMS-coated CPC electrodes, considering the typical thickness of a regular shirt. The electrode attachment configurations are described in Figure 6a. The EEG measurement performance was validated by an eye open (EO) and eye close (EC) experiment. Alpha band (8~12 Hz) brainwaves were expected to increase when the eyes were closed. Six voluntary subjects (six males, average age = 33.7) participated in the experiment. A subject was asked to use a chinrest and watch a red dot in a monitor to fix the eye in EO condition. A sound alarm advised the subject to open his eyes for 30 s and close his eyes for 30 s and to repeat this one time. This experiment was performed with each CPC and gel electrode. Figure 6b–d show one representative result. Figure 6b shows the clear difference between EO and EC with both conventional and coated CPC electrodes. A high peak on the 30 s point corresponded to eye-closing movement. Occasional eye movements could be observed before 30 s. After 30 s, higher frequency signals due to the alpha wave were visually observable. A closeup of the EO and EC transition was provided in Figure S14. Figure 6c shows a spectrogram of the EEG data and the power spectrum with gel electrode data. Figure 6d shows PDMS-coated CPC electrode data as well. The average alpha band power difference of EC and EO states of the six subjects is displayed in Figure 6e. The PDMS-coated CPC electrode shows a similar difference in a comparison with the gel electrode. However, the variation was higher on the CPC electrode, potentially due to a less stable skin contact. The power spectrum of all subjects is shown in Figure S15.

To illustrate long-term stability and the potential for sleep monitoring, one subject took a brief nap during the study. This subject initially laid down for 10 min with their eyes open and then took a 30-min nap. Figure 6f displays a spectrogram of 40-min recordings. The red dashed box highlights a higher frequency, which corresponds to sleep spindles. In Figure 6g, the purple box represents a sleep spindle automatically detected by the manufacturer’s program. Additionally, Figure S16 displays the automatic sleep stage classification results during the eye-closed time. This experiment demonstrates the feasibility of using CPC electrodes for sleep monitoring applications.

## 4. Discussion and Conclusions

This study presented a comprehensive exploration of a CNT–paper composite for biopotential measurements. To ensure biocompatibility, various polymer materials, including PDMS, Eco-flex, PI, and PU, were applied to CPC electrodes and evaluated for EOG measurements. Among these coatings, PDMS and Eco-flex demonstrated superior SNRs compared to others. While SNR values for PDMS- and Eco-flex-coated electrodes were slightly lower than conventional gel electrodes, they remained sufficiently functional for biopotential measurements. However, PDMS has a higher MVTR with a lower resistance compared to Eco-flex, minimizing the susceptibility to noise and motion artifacts during measurements. Based on the results, we decided to use PDMS as the biocompatible coating material in our experiments. The study showcased the measurement of ECG for monitoring HR, RR, and HRV through signal processing with PDMS-coated CPC electrodes. Additionally, EEG measurements were conducted and analyzed to demonstrate their potential for sleep monitoring. These findings highlighted that the coated CPC electrodes, when integrated into a compact system, enabled safe, reliable, and real-time biopotential recording in everyday scenarios, encompassing EOG, ECG, and EEG. Unlike gel-based electrodes, the CPC electrodes did not generate any skin irritation. The CPC electrodes presented in this study hold significant promise for applications in daily life healthcare monitoring.

## Data Availability

Data are contained within the article.

## Supplementary Materials

The following supporting information can be downloaded at: https://www.mdpi.com/article/10.3390/s23249727/s1, Figure S1: Fabrication of CPC electrodes step-by-step; Figure S2: Photos showing both the front and back of CPC electrodes; Figure S3: Photo of the BioRadio with detailed specifications; Figure S4: Photos of the locations for wearing electrodes; Figure S5: Photo of SOMNOtouch RESP with detailed specifications; Figure S6: Photos of custom-made frames to produce a thin polymer film; Figure S7: Photos of the CPC resistance measurement; Figure S8: Measurement of the CPC surface with digital multimeters; Figure S9: Photographs of a skin rash to show how long a rash lasts; Figure S10: Detailed SNR values; Figure S11: Comparison of EOG signals using two types of electrodes; Figure S12: Comparison of ECG signals using two types of electrodes; Figure S13: Graph of spline interpolated R-peaks; Figure S14: Comparison of EEG signals using two types of electrodes; Figure S15: All six subjects’ eye open and eye close experiment results used in Figure 6e); Figure S16: (a) EEG spectrogram of Figure 6f eye closed condition; (b) automatic sleep stage classified by manufacturer program; Table S1: Detailed resistance of the four CPC sheets; Table S2: Detailed CPC electrode resistance; Equation (S1): Equations which are used for calculating heart rate variability metrics; Video S1: ECG measurement demonstration.
