# Supplementary material for: Continuous Biopotential Monitoring via Carbon Nanotubes Paper Composites (CPC) for Sustainable Health Analysis
Source: Sensors (Basel). 2023 Dec 9;23(24):9727. doi: 10.3390/s23249727 (PMC10748204; doi:10.3390/s23249727)
Supplement: Supplementary file 1 [file sensors-23-09727-s001.zip › sensors-2728088-supplementary.pdf]

CPC SI

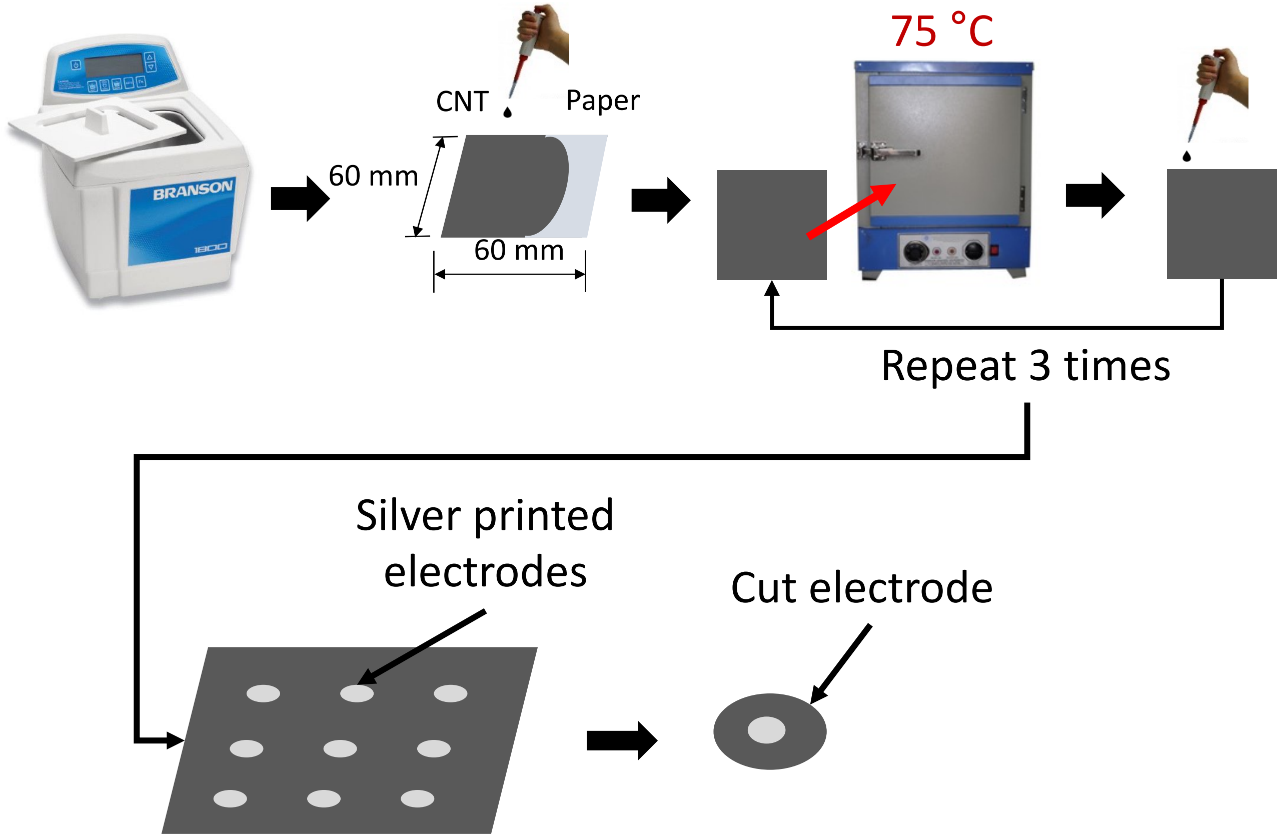

Figure S1. Fabrication of CPC electrodes step-by-step

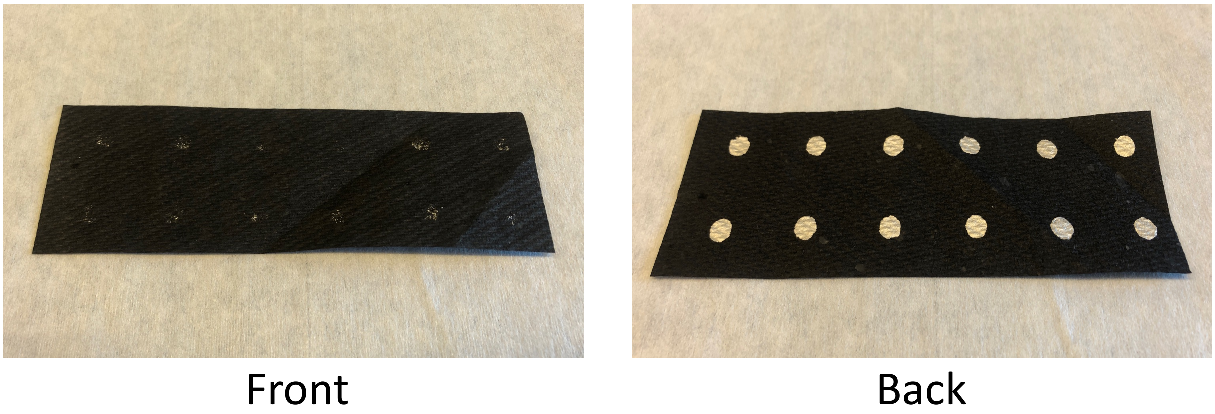

Figure S2. Photos showing both the front and back of CPC electrodes

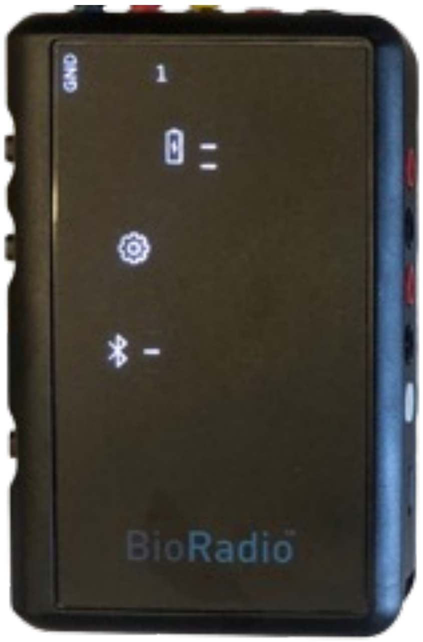

Specifications	Value
Dimensions	10 x 6 x 2 cm (4oz)
Wireless Connection	Bluetooth Classic / Low Energy
Transmission Range	30 meters
Differential or Single-ended channels	4 or 8
Sampling rate	250 Hz
Recording Time Battery Life	8 hours

Figure S3. Photo of BioRadio with detailed specifications of BioRadio

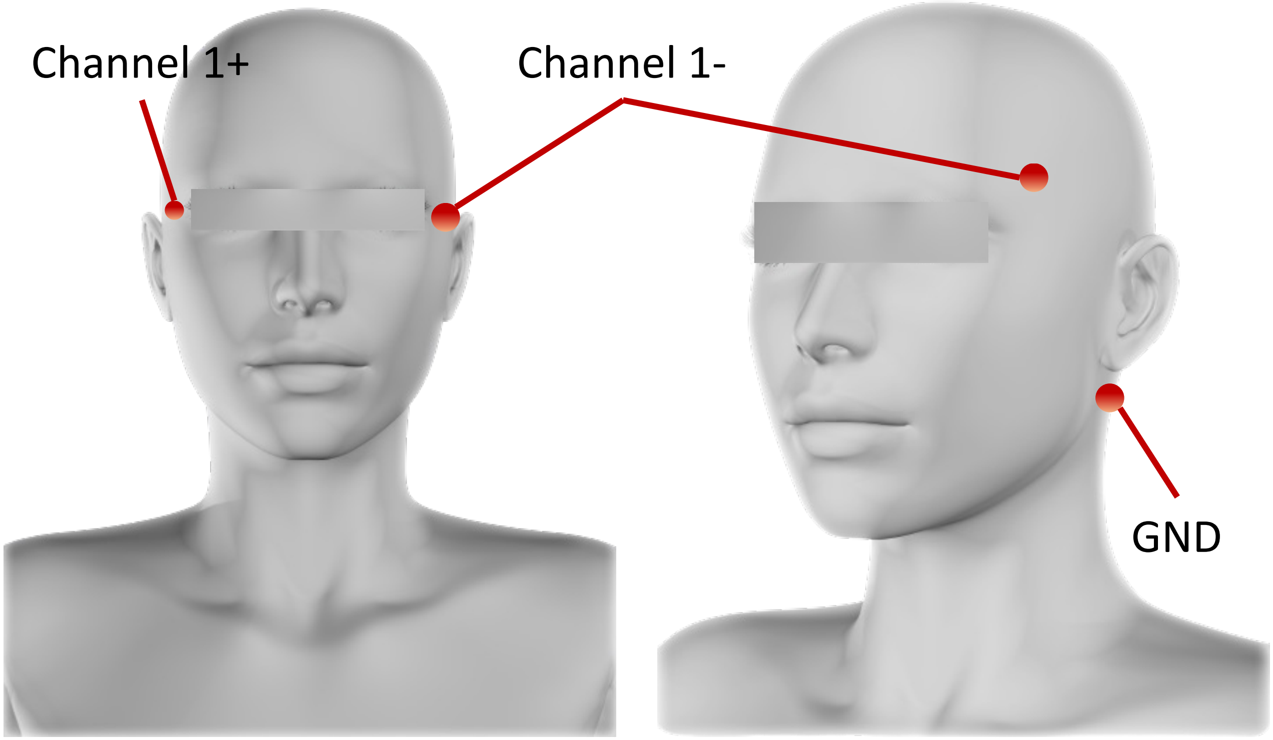

Figure S4. Photos of location for wearing electrodes

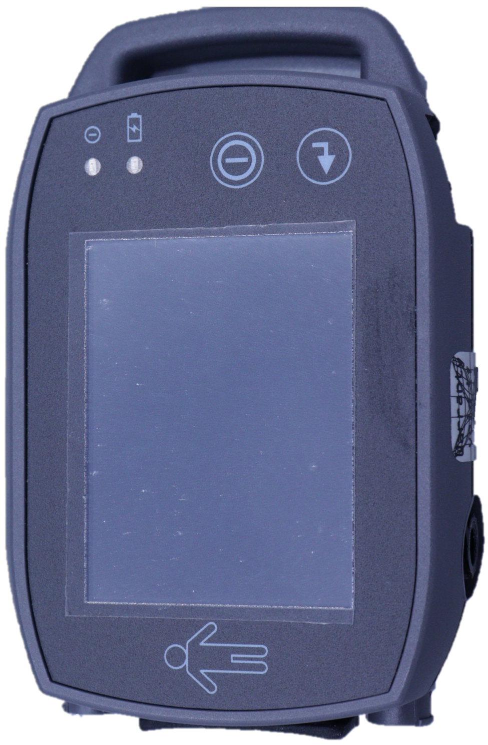

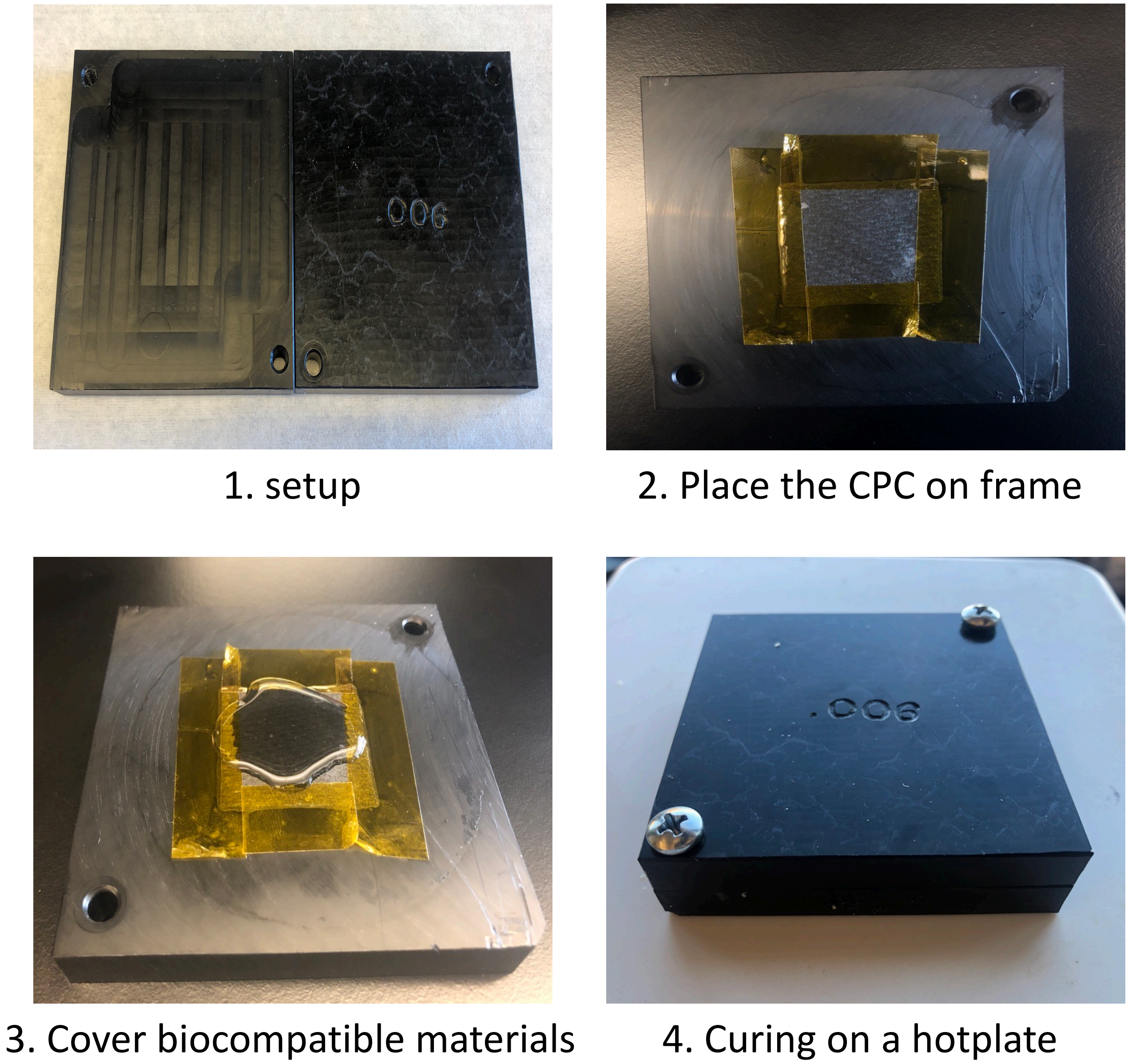

Figure S6. Photos of custom-made frames to produce thin polymer film

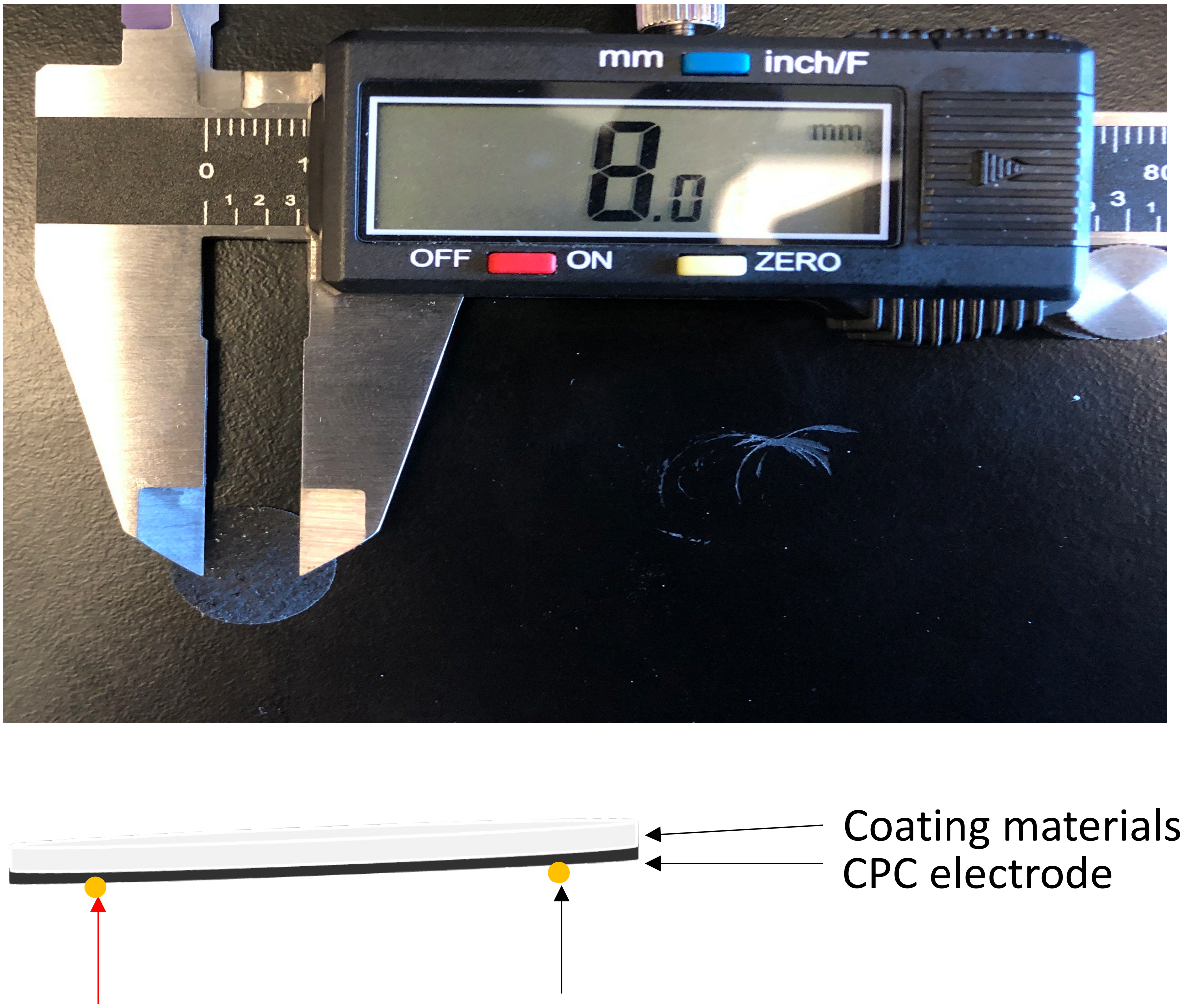

Figure S7. Photos of CPC resistance measurement (Bottom side)

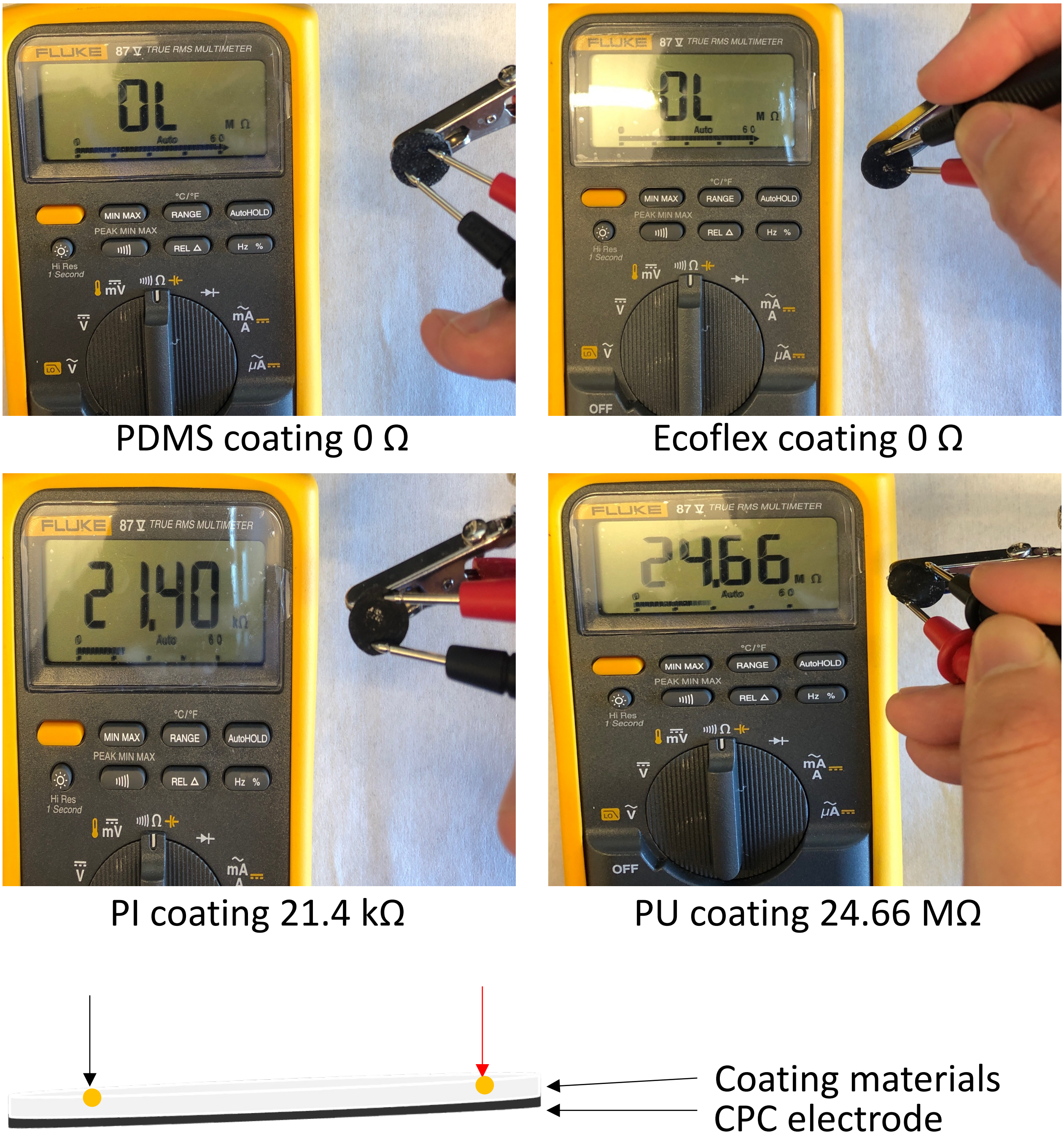

Figure S8. Measurement of CPC surface with digital multimeters (Top side)

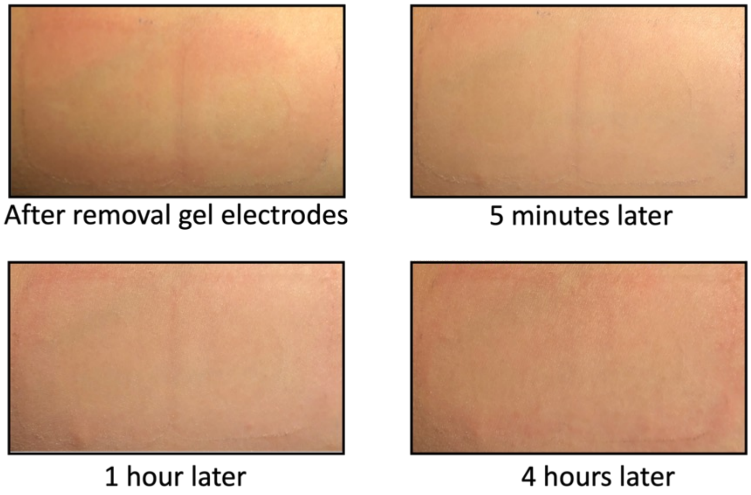

Figure S9. Photographs of skin rash to show How long a rash lasts.

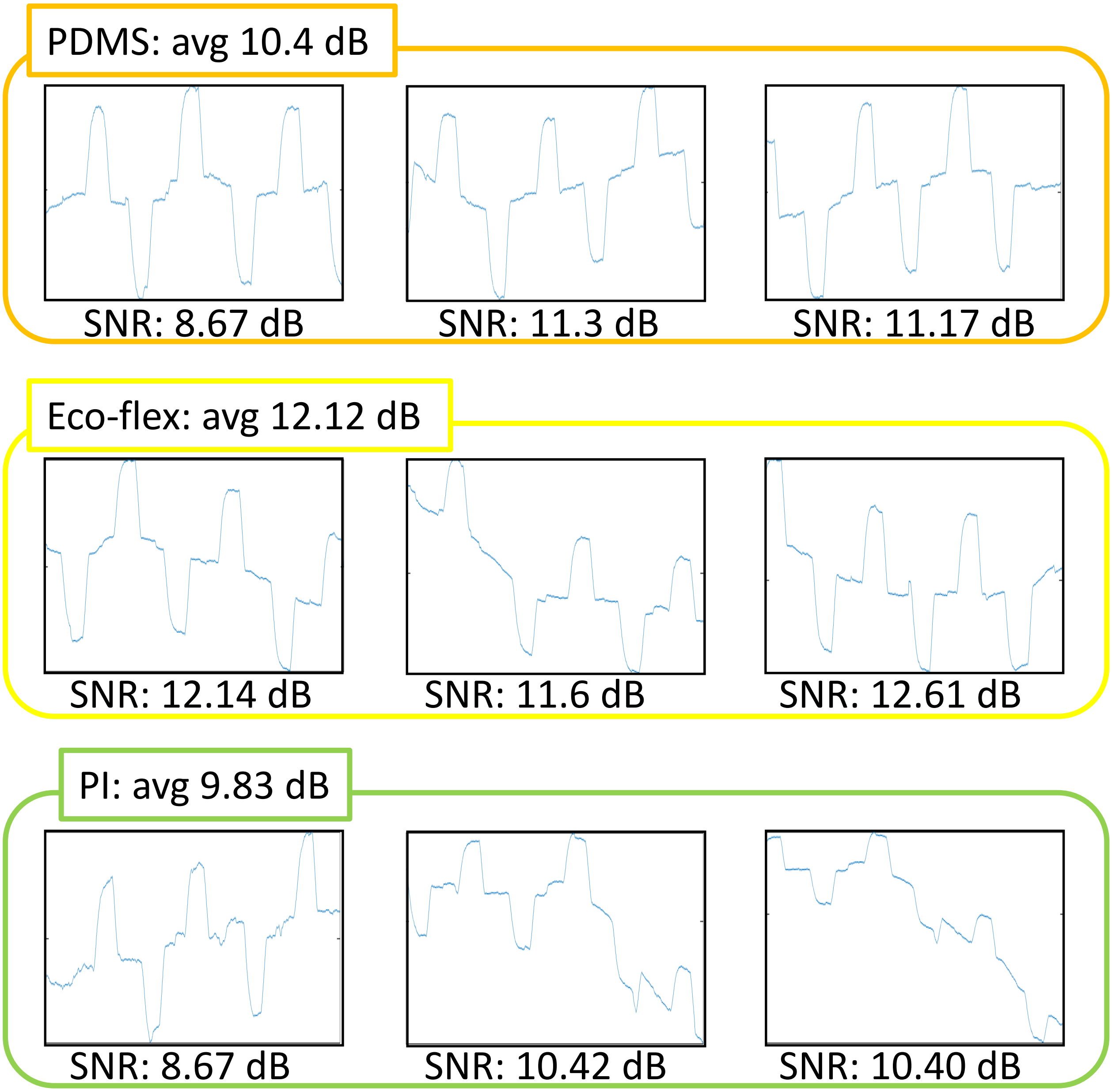

Figure S10. Detailed SNR values

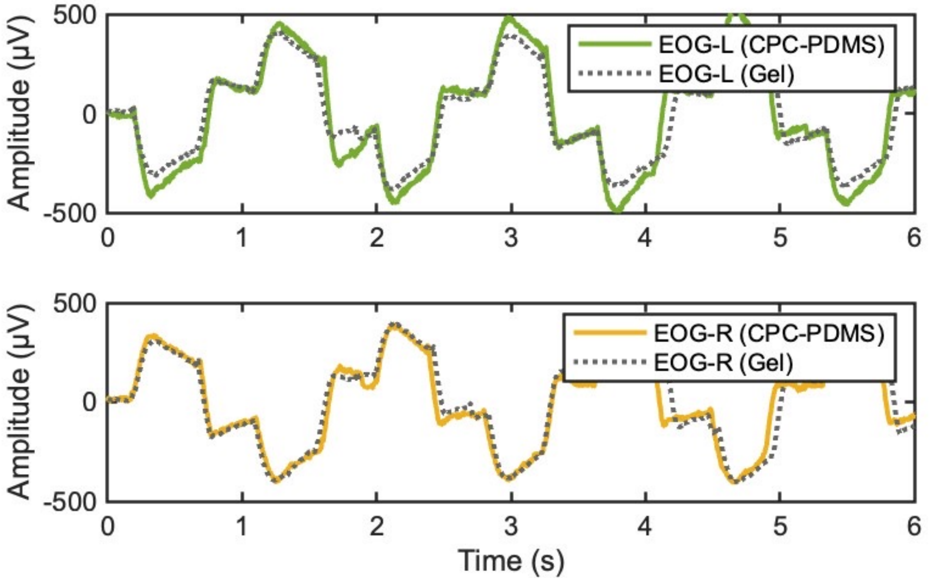

Figure S11. Comparison of EOG signals using two types of electrodes

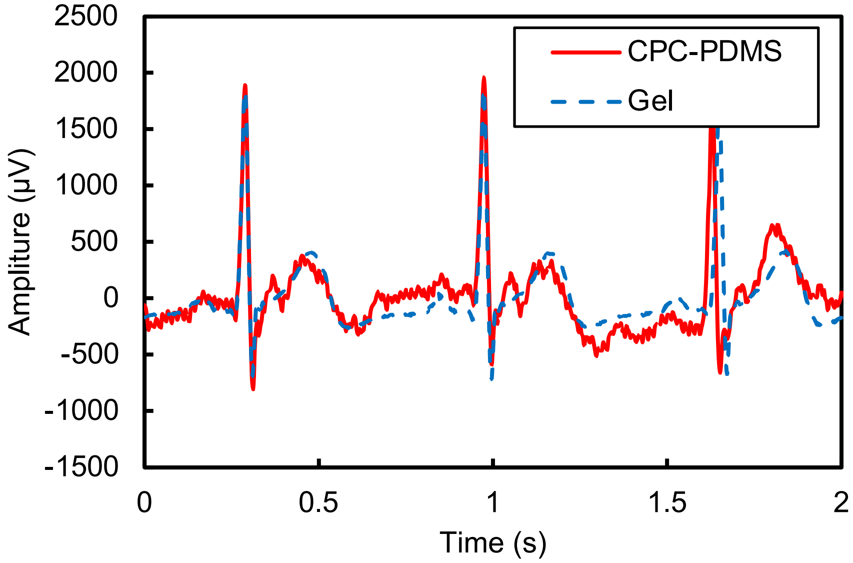

Figure S12. Comparison of ECG signals using two types of electrodes

a)

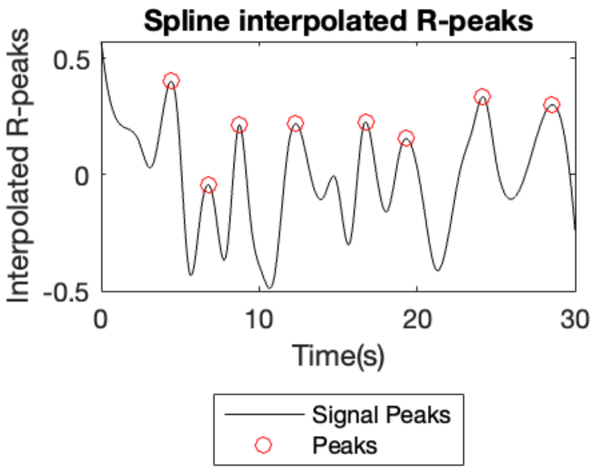

Figure S13. Graph of spline interpolated R-peaks

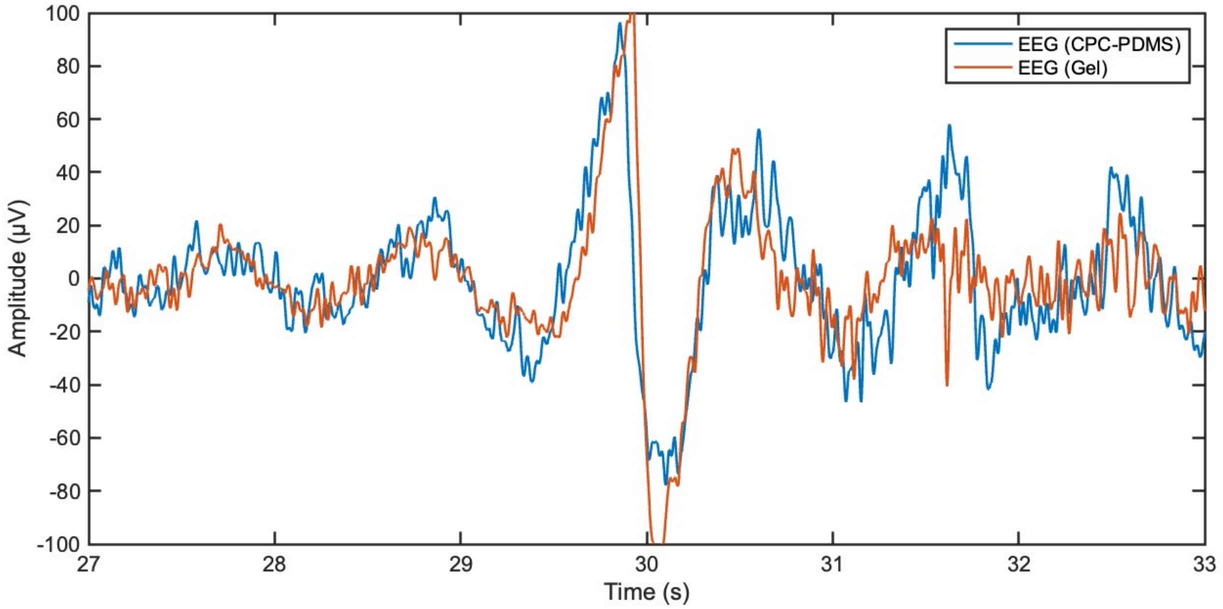

Figure S14. Comparison of EEG signals using two types of electrodes

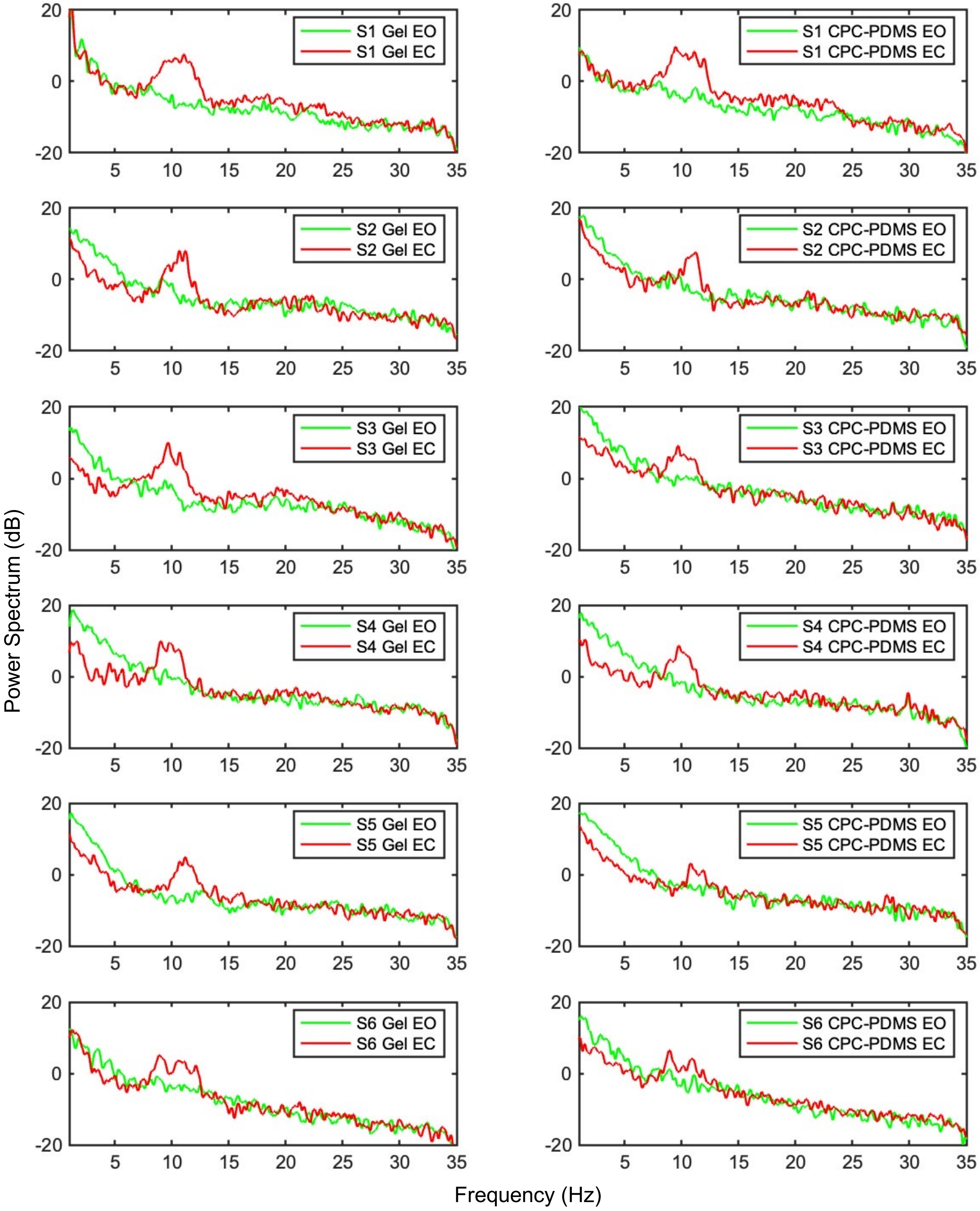

Figure S15. All six subjects' eye open and eye close experiment results used in Figure 6e)

a)

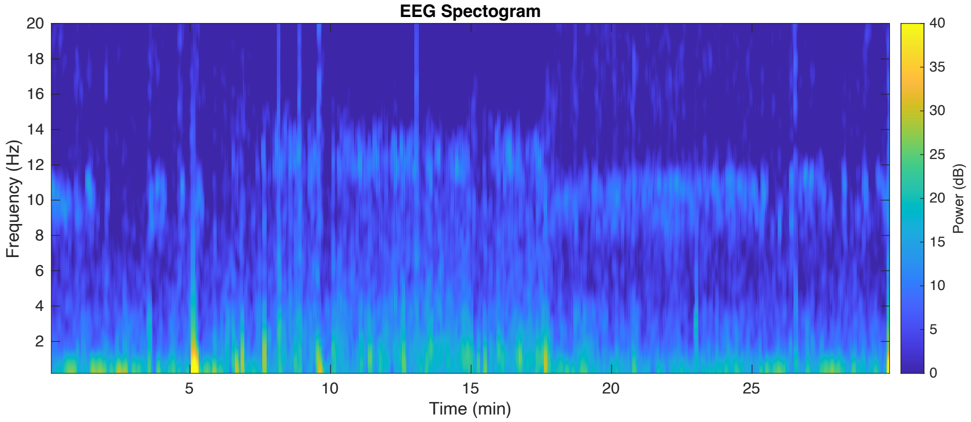

b)

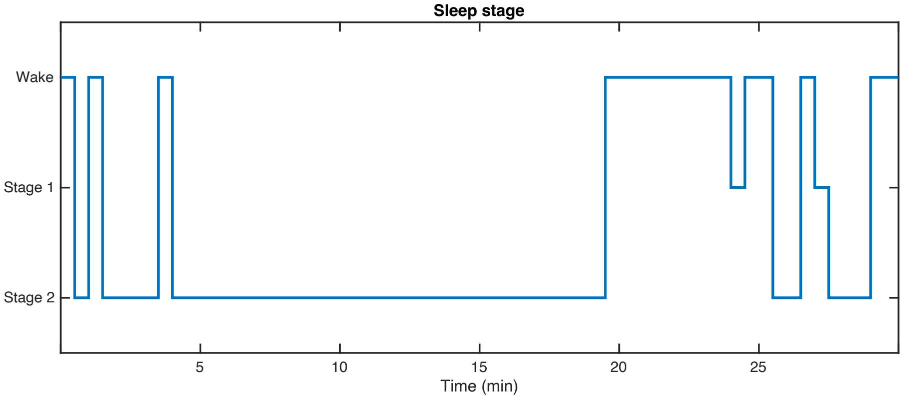

Figure S16. a) EEG spectrogram of Figure 6f eye closed condition, b) Automatic sleep stage classified by manufacturer program

Table S1.

	Current (mA)	Voltage (V)	Resistance (Ω/□)
Sheet 1	6.84	0.528	350
	6.42	0.525	370
	6.18	0.53	388
	6.72	0.666	449
	6.32	0.563	404
Sheet 2	5.69	0.725	577
	6.81	0.532	354
	6.13	0.522	386
	5.61	0.552	446
	5.15	0.445	391
Sheet 3	6.32	0.495	355
	6.31	0.511	367
	6.21	0.648	473
	5.52	0.425	349
	5.49	0.562	464
Sheet 4	6.1	0.593	440
	6.55	0.637	441
	5.82	0.582	453
	5.03	0.526	474
	5.33	0.548	466
		STD	58.48
		Average	420

Table S2.

Coating type	Resistance (kΩ)
Bare CPC	0.89
	0.9
	0.9
	0.83
	0.82
	1.0
**Average**	**0.89**
PDMS 0.15 mm	3.2
	3.7
	3.3
	3.1
	4.0
	3.0
**Average**	**3.83**
PDMS 0.5 mm	3.5
	4.8
	2.6
	3.4
	3.6
	2.9
**Average**	**3.46**
Eco-flex 0.15 mm	8
	7.4
	6.6
	7.2
	8.4
	8.2
**Average**	**7.63**

Equation S1. Equations which is used for calculating heart rate variability metrics. All equations below are from

$$\mathrm{RR} = R - R\ interval, n = total\ number\ of\ the\ RR, m = average\ of\ entire\ RR$$

$$SDRR = \sqrt{\frac{(RR_1 - m)^2 + (RR_2 - m)^2 + \cdots + (RR_n - m)^2)}{n}}$$

$$RMSSD = \sqrt{\frac{(RR_1^2 + RR_2^2 + \cdots + RR_n^2)}{n}}\ CVRR = \frac{SDRR}{m}$$
